# Positive mixture effects in pine–oak forests during drought are context‐dependent

**DOI:** 10.1111/plb.70030

**Published:** 2025-05-07

**Authors:** G. Schmied, J. Kappen, M. del Río, W. K. Moser, M. J. Gundale, T. Hilmers, D. Ambs, E. Uhl, H. Pretzsch

**Affiliations:** ^1^ Professorship of Tree Growth and Wood Physiology, Department of Life Science Systems, TUM School of Life Sciences Technical University of Munich Freising Germany; ^2^ Chair for Forest Growth and Yield Science, Department of Life Science Systems, TUM School of Life Sciences Technical University of Munich Freising Germany; ^3^ Department of Forest Ecology and Management Swedish University of Agricultural Sciences Umeå Sweden; ^4^ Instituto de Ciencias Forestales ICIFOR‐INIA, CSIC Madrid Spain; ^5^ USDA Forest Service Rocky Mountain Research Station Flagstaff Arizona USA; ^6^ Bavarian State Institute of Forestry (LWF), Bavarian State Ministry of Food, Agriculture and Forestry (StMELF) Freising Germany; ^7^ Sustainable Forest Management Research Institute iuFOR University of Valladolid Valladolid Spain

**Keywords:** niche complementarity, pine–oak forests, recurrent drought stress, species mixture

## Abstract

- The increasing severity and frequency of droughts will play a pivotal role in shaping future forest ecosystems worldwide. Trees growing in mixtures are thought to be less susceptible to drought stress, but evidence for such positive admixture effects remains limited. This study examines how interspecific neighbourhood structures affect the growth responses of pine and oak species under recurrent drought stress in two contrasting forest ecosystems.
- We sampled naturally occurring, unmanaged mixed stands of Gambel oak (*Quercus gambelii*) and ponderosa pine (*Pinus ponderosa*) in semi‐arid Arizona, USA, and pedunculate oak (*Quercus robur*) and Scots pine (*Pinus sylvestris*) in sub‐humid Bavaria, Germany. Tree growth responses to recurrent drought events were assessed across a wide gradient of species admixture.
- Species admixture significantly influenced tree growth responses to drought stress, but the effects varied by species and forest ecosystem. In semi‐arid Arizona, increasing species admixture buffered trees, especially Gambel oak, against drought stress. In sub‐humid Bavaria, the effects of species admixture on pedunculate oak and Scots pine were more variable.
- Our findings emphasize the positive mixture effects in semi‐arid environments, likely due to distinct niche complementarity and facilitation. Under sub‐humid conditions, the effects were less consistent, aligning with the stress‐gradient hypothesis. This study provides valuable insights into the complex dynamics of pine–oak interactions under drought stress and emphasizes the relevance of complementary species admixtures for climate‐smart forest management in the face of climate change.

## Graphical abstract

Mixture effects in drought‐stressed pine‐oak forests are context‐dependent, with Gambel oak and ponderosa pine benefiting from their complementarity under semi‐arid conditions.

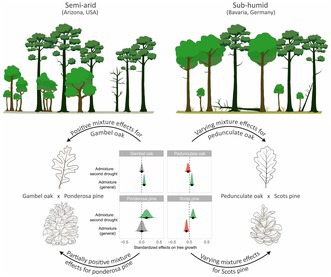

## INTRODUCTION

The relationship between biodiversity and ecosystem functioning has been extensively explored across various ecosystems, from grasslands to forests. Many studies in grassland ecosystems have shown that higher species diversity can lead to increased productivity, stability, and resilience (Tilman *et al*. 2001; Isbell *et al*. 2015; Wang *et al*. 2019). These benefits have been found to strengthen over time (Cardinale *et al*. 2007) and have been attributed to niche complementarity (Hector *et al*. 2010; Zuppinger‐Dingley *et al*. 2014), where interacting species with different functional traits utilize resources more efficiently. Positive effects of diversity on ecosystem functioning in forests have been considered less often, and the evidence for systematic positive mixing remains sparse or inconclusive (Grossiord 2020; Looney *et al*. 2021; Depauw *et al*. 2024). Nevertheless, tree species richness has been associated with increased productivity (Jactel *et al*. 2018), light uptake (Depauw *et al*. 2024), nutrient availability (Schmidt *et al*. 2015), growth efficiency (Hilmers *et al*. 2024), or drought resistance (Pretzsch *et al*. 2013). Similarly, the mechanisms behind these positive effects depend on the functional traits and niche partitioning of the interacting tree species (Fichtner *et al*. 2017, 2020; Depauw *et al*. 2024), which can lead to complementarity effects through facilitation or reduced competition (Ammer 2019). For instance, mixing two species with different morphological crown characteristics results in increased stand density (Pretzsch & Biber 2016), crown coverage (Pretzsch 2014) and crown complementarity (Williams *et al*. 2017; Hilmers *et al*. 2024) compared to monospecific stands, which facilitate resource acquisition through spatial niche differentiation (Pretzsch 2014) and stabilizes productivity (del Río *et al*. 2022).

Against the backdrop of climate change, increasing intensity and frequency of droughts are becoming a major concern for forest ecosystems (Anderegg *et al*. 2015). Recent observations indicate that the effects of recurring drought stress accumulate and lead to significant negative legacies (Peltier & Ogle 2019), along with declines in growth, losses of vitality, and increases in mortality in many temperate forests (Anderegg *et al*. 2013; Schmied *et al*. 2023). However, effective strategies to attenuate these effects remain elusive. As it has been repeatedly shown that drought impacts on tree growth are less severe in mixtures (Fichtner *et al*. 2020; Steckel *et al*. 2020; Pardos *et al*. 2021), buffering drought stress by increasing tree species diversity is considered a viable forest management strategy (Brang *et al*. 2014; Messier *et al*. 2022). Although some indications of positive mixing effects exist, several studies have found little, none or negative effects, calling into question the general benefits of mixtures under drought stress and its systematic implications on forest management (Grossiord *et al*. 2014; Grossiord 2020; Haberstroh & Werner 2022; Mas *et al*. 2026). The assumption that mixing different tree species could be beneficial is based on the concept that drought impacts are alleviated by adapted resource allocation, facilitation and selection effects (Ammer 2019; Grossiord 2020). However, Grossiord (2020) emphasized that in contrast to grassland ecosystems, mixture effects in forest ecosystems are more context‐dependent. According to the stress‐gradient hypothesis (SGH), plant interactions tend to shift from being mainly competitive to becoming more facilitative as environmental stress increases (Bertness & Callaway 1994), depending on the species' functional traits (Maestre *et al*. 2009). This is supported by findings from Grossiord *et al*. (2014) that positive mixture effects in forests are more pronounced in drought‐prone environments. Thus, cross‐ecosystem studies comparing similar tree species compositions under contrasting climate conditions could provide valuable insights into mixture effects, considering the implications of the SGH hypothesis. Here, we directly address this theory by focusing on pine–oak mixtures with varying functional traits across highly contrasting environments (sub‐humid vs. semi‐arid climates) since previous studies have shown evidence of positive mixture effects for these genera in particular (Bello *et al*. 2019; Pretzsch *et al*. 2020; Steckel *et al*. 2020).

Pine and oak species are widely distributed in the northern hemisphere, with species of both genera inter‐mixing across different continents and forest ecosystems. Mixed pine–oak forests can be found from Eastern Asia (Chai *et al*. 2016), throughout Europe (Pretzsch *et al*. 2020), to eastern (Hart *et al*. 2024) and western (Poulos 2009) USA, covering a large gradient of different climatic and edaphic conditions. These mixtures often occur in forests that grow on well‐drained, sandy soils or other water‐limited regions (Poulos 2009), which in North America, for example, are often characterized by a pronounced seasonal distribution of precipitation and suppression by fire (Guiterman *et al*. 2015; Hart *et al*. 2024). Their ability to tolerate drought stress, to grow on nutrient‐poor soils, or to thrive after fires by quickly re‐sprouting and regenerating enables them to occupy sites that are unfavourable to more mesic species that would otherwise outcompete them (Nowacki & Abrams 2008; Poulos 2009). Even though species of both genera often co‐occur, they differ in their functional traits and occupy different niches. Oaks have larger, often deciduous leaves, rely on wider vessels with higher leaf‐specific hydraulic conductivity for water transport (McCulloh *et al*. 2010), and are usually more shade‐tolerant than co‐existing pines (Hart *et al*. 2024). Under drought stress and increased evaporative demand, they tend to have a more negative leaf water potential without risking severe damage from cavitation, indicating anisohydric water use (Zweifel *et al*. 2007). In contrast, the more light‐demanding pines feature small, evergreen needles, narrower tracheids for water movement, and more drought‐sensitive stomata, highlighting the divergent physiological strategies between the two genera (Zweifel *et al*. 2007; Klein *et al*. 2013). Moreover, pine and oak species differ in the morphology and plasticity of their crown (Pretzsch 2014) and roots (Correia *et al*. 2018), particularly evident when they grow together in mixture, (Pretzsch 2014; Correia *et al*. 2018; del Río *et al*. 2019), which further emphasizes their complementary niches. In some regions, pine–oak mixtures display a distinct forest structure, with oaks forming a secondary layer beneath the pine canopy, indicating an even more pronounced spatial niche differentiation (Abella 2008; Hart *et al*. 2024). In essence, pine and oak species frequently co‐exist in the same environments, but exhibit large differences in functional traits and niche preferences in forests throughout the northern hemisphere.

We utilized the occurrence of pine–oak mixtures in highly different forest ecosystems to investigate mixture effects under recurrent drought stress. While previous studies focused mainly on single drought years and managed forests within the same region or forest ecosystem, we directly addressed this knowledge gap by selecting unmanaged forest stands of ponderosa pine and Gambel oak in semi‐arid Arizona, USA, and Scots pine and pedunculate oak in sub‐humid Bavaria, Germany. Relying on a dendroecological approach, we aimed to identify whether there are general positive growth effects under unfavourable conditions due to increased species admixture, and whether these effects persist under recurrent drought stress and in different forest ecosystems. In more detail, we derived the following research questions:

1. Do pine and oak species benefit from an interspecific neighbourhood under drought stress in terms of their growth?
2. Are there persistent mixing effects under recurring drought stress?
3. Are mixture effect patterns consistent across contrasting forest ecosystems with different pine and oak species?

## MATERIALS AND METHODS

### Study site selection and regional characteristics

**Fig. 1:**
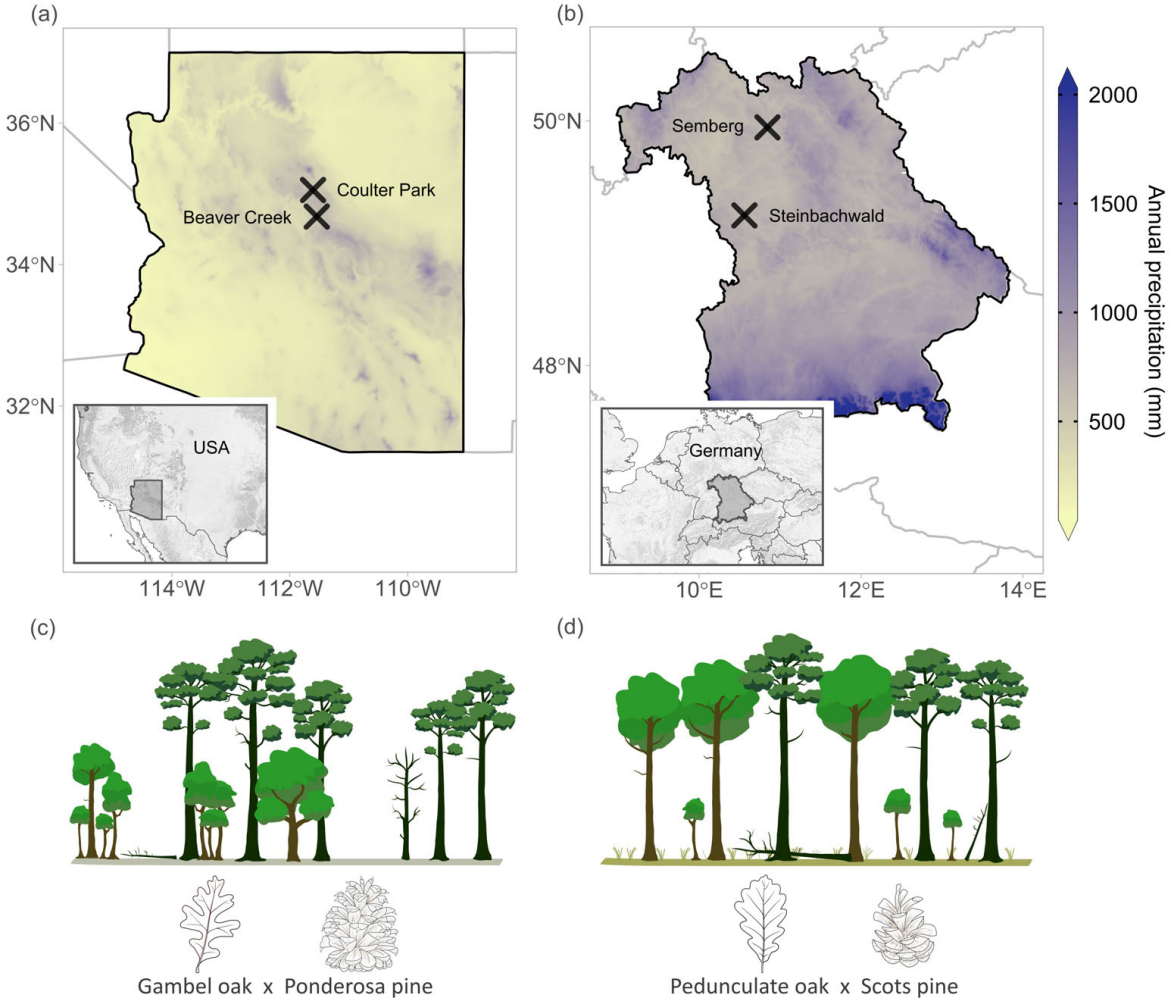
Average annual precipitation from 1991 to 2020 in Arizona, USA (a) and Bavaria, Germany (b), including the respective study site locations (black crosses). Schematic representations of the corresponding characteristic forest structures are shown in (c) and (d).

Between May and November 2023, we selected and sampled four sites, two in Arizona, USA, and two in Bavaria, Germany. The two contrasting regions were selected for their naturally occurring pine–oak mixtures in contrasting climate conditions (sub‐humid versus semi‐arid; see Table 1, Fig. 1). Suitable sites offered: (i) a broad gradient of different mixture proportions (from pure to mixed conditions), (ii) a basal area share of other species <10%, and (iii) no tree harvesting activities for at least the past 50 years. The two selected forest stands in Arizona were dominated by Gambel oak (*Quercus gambelii* Nutt.) and ponderosa pine (*Pinus ponderosa* subsp. *brachyptera* Engelm.) and were situated in the Fort Valley Experimental Forest near Flagstaff (site ‘Coulter Park’) and the Coconino National Forest near the Mogollon Rim (site ‘Beaver Creek’). The forests in Bavaria, on the other hand, were in central (site ‘Steinbachwald’) and lower Franconia (site ‘Semberg’) and were dominated by pedunculate oak (*Quercus robur* L.) and Scots pine (*Pinus sylvestris* L.). Each selected site originated from natural regeneration and developed under relatively natural conditions. However, it should be noted that while no tree harvesting activities have occurred at the Arizona sites, historical land use includes cattle grazing near Beaver Creek approximately a century ago. In addition, both repeated wildfires and prescribed burns have been integral to the natural dynamics of these forests, contributing to the maintenance of their open stand structure. The unmanaged Bavarian sites were located in otherwise regularly managed forests. In general, the southwestern USA has a bimodal precipitation regime, with most water coming from the melting snowpack in spring and the North American Monsoon (NAM) in summer, separated by hyper‐dry periods, with extremely high vapour pressure deficits (VPD) (Bailey *et al*. 2023). This contrasts with the mild climate in Central Europe (see Fig. 1a,b), which offers favourable conditions for tree growth due to cyclonic rain events at all times of year (Ellenberg & Leuschner 2010). However, during the past decades, Central Europe has experienced increased occurrence of droughts (Rakovec *et al*. 2022). Thus, the trees growing at the semi‐arid sites in the USA are regularly and heavily water‐limited during summer, while those growing under subhumid conditions in Central Europe are generally light‐limited, and only water‐limited during drought periods. The annual mean temperature in Arizona was 8.5–8.9°C, and annual mean precipitation was 599–625 mm. For Bavaria, the annual temperature and precipitation were 8.7–9.3°C and 704–723 mm, respectively (see Table 1 for overview). The sites in northern Arizona were on shallow, loamy, clay soils with low water‐holding capacity, barely any humus layer, and <100 cm to bedrock material (Leptosols), while the Bavarian sites were on relatively sandy (Cambisol, Semberg) and more fertile loamy soils with periodically wet topsoil (Stagnosol, Steinbachwald).

**Table 1:** Overview of the site characteristics.

site name	species	latitude (N)	longitude (E)	temperature (°C)^a^	precipitation (mm year^−1^)^a^	aridity index^b^	basal area (m^2^ ha^−1^)^c^	deadwood basal area (m^2^ ha^−1^)^c^	elevation (m a.s.l.)	soil type
Coulter Park (USA)	Gambel oak & ponderosa pine	35°03′16″	−111°36′17″	8.5 (15.4)	625 (88)	0.49 (semi‐arid)	52.5 ± 16.8	3.13 ± 4.83	2190	Leptosol
Beaver Creek (USA)	Gambel oak & ponderosa pine	34°44′29″	−111°30′15″	8.9 (15.7)	599 (86)	0.47 (semi‐arid)	43.4 ± 15.9	5.72 ± 9.18	2055	Leptosol
Semberg (GER)	Pedunculate oak & Scots pine	49°56′51″	10°50′35″	9.3 (16.5)	704 (210)	0.89 (sub‐humid)	48.0 ± 8.31	4.91 ± 3.29	360	Cambisol
Steinbachwald (GER)	Pedunculate oak & Scots pine	49°13′39″	10°33′30″	8.7 (15.7)	723 (229)	1.00 (sub‐humid)	41.1 ± 9.23	3.42 ± 2.75	470	Stagnosol

^a^ Based on average annual values for the period 1991–2020. Average values for late spring and early summer (May, June and July) are given in brackets.

^b^ Average ratio between annual precipitation (P) and potential evapotranspiration (PET). Semi‐arid = 0.20 ≤ P/PET <0.50; Sub‐humid = 0.65 ≤ P/PET ≤1.00.

^c^ Based on measurements within the biogroups. Mean ± SD is presented. Basal area measurements in Arizona do not represent the entire forest stand due to the uneven spacing between trees (trees often occur in dense clusters). The actual basal area is lower than that of the sites in Bavaria.

The contrasting conditions lead to different forest dynamics and structures. The mixed forests of ponderosa pine and Gambel oak were characterized by relatively open stands, with Gambel oak occurring in unevenly spaced clumps, dense thickets, or as single trees (Abella 2008), forming a second layer underneath the ponderosa pine canopy (see Fig. 1c)—typical characteristics of this vegetation type (the so‐called ‘pine–oak belt’). In contrast, Scots pines and pedunculate oaks co‐occurred in the upper layer, were more evenly distributed across the stand, and formed a closed canopy with a high stand density (Fig. 1d). An overview of species‐specific differences at the study sites is given in Fig. S1, revealing similar diameters for all four species but differing heights, living crown ratios and crown projection areas.

### Tree selection and field sampling

We selected 30 oak and 30 pine trees at each of the four sites, resulting in a total of 240 trees (from here‐on called target trees). Only healthy (co‐)dominant trees that showed no apparent signs of pest, fungal or parasite infestation (e.g. dwarf mistletoe on ponderosa pine) were chosen. Gambel oaks, both as solitary trees and in dense thickets or clumps, were selected to represent the full range of structural types found in the forests. We visually estimated the individual mixture proportions among direct competitors in a 7‐m radius before selection to achieve broad coverage of a mixture gradient within each site (from intraspecific to interspecific competition). For this purpose, each target tree was classified into one of three categories based on its neighbourhood structure: (i) <25%, (ii) 25%–75%, or (iii) >75% basal area shares of other species. An equal number of 10 target trees were selected for each category per site and species. All target trees were located within a 2‐ha area and at least 50 m from the forest edge.

For all target trees, we measured diameter at breast height (dbh_1.3m_; cm), total tree height (m), crown base height (m), and crown radii in eight directions (N, NE, E, SE, S, SW, W, NW; m), using a Vertex ultrasonic hypsometer (Haglöf, Madison) and girth tapes. Within a radius of 7 m around each target tree (= 154 m^2^), all neighbouring trees above a dbh_1.3m_ of 1 cm were recorded (= 4032 trees), whereby tree species, dbh_1.3m_, as well as distance and angle to the respective target tree were measured. Standing and downed dead trees were also recorded (= 672 trees). In addition, we cored each target tree from the northern and eastern cardinal directions using a 5.15 mm Pressler increment borer. Overall, we sampled 480 cores, which were air‐dried and mounted on wooden support pieces.

### Tree‐ring data

All tree cores were sanded with progressively finer grit sandpaper (120–800) to enhance visibility of annual tree‐ring boundaries. Tree‐ring widths were measured to the nearest 0.01 mm, following two different approaches. As we cored the trees in Arizona during the growing season of 2023, the growth ring of 2023 was not yet fully developed. We therefore excluded the growth in 2023 from our measurements. Tree cores from Germany were examined using the digital positioning table LINTAB 5 and the software TSAPWIN (both Rinntech, Heidelberg). In contrast, the tree cores from the USA were measured from scanned images using the software CooRecorder 9.8.1 and CDendro 9.8.1 (Cybis, Sweden). The different measurement approaches were chosen for logistical reasons and are expected to yield equivalent data. All tree‐ring series were visually cross‐dated, considering anomalous growth years that are consistent for any individual site (Schweingruber *et al*. 1990). Due to the bimodal cambial activity of ponderosa pine trees under a monsoon climate (Morino *et al*. 2021), the formation of false rings (i.e. intra‐annual density fluctuations) complicated the measurements. We relied on published chronologies from the Coconino National Forest (Brice *et al*. 2021) and the more easily measurable Gambel oaks as a reference to allow correct dating. Finally, we conducted statistical cross‐dating to identify potential dating errors using skeleton plots and correlation analyses. Further, calculations of the mean inter‐series correlation (rbar), the expressed population signal (eps), and sign test (*Gleichläufigkeit*, glk) provided additional insight into the similarity of growth patterns and how well the population was represented by the sampled trees (Speer 2010). We transformed the annual ring width measurements into basal area increments (bai) using the formula ${\mathrm{bai}}_{t}=\pi \times \left(r_{t}^{2}-r_{t-1}^{2}\right)$, where r represents the tree radius at dbh_1.3m_ and t the respective year, as it is assumed that the bai better reflects the actual biomass increment of the trees (Biondi & Qeadan 2008). The two bai series per tree were averaged to obtain an individual growth series for each target tree and then converted into dimensionless ring‐width indices (rwi) by de‐trending them with a cubic‐smoothing spline with a 50% frequency cut‐off at 30 years, which minimizes low‐frequency growth variations associated with age‐ and size‐related growth trends. Additionally, we built a chronology for each site and species by averaging all respective rwi using Tukey's biweight robust mean for the assessment of correlations between climate and growth (see section 2.5, Fig. S2).

### Climate data

We obtained meteorological data on time series of monthly precipitation (mm) and mean, minimum, and maximum temperature (°C) for all study sites. For the two sites in Arizona, we extracted interpolated PRISM estimates with a resolution of 4 × 4 km (PRISM Climate Group, https://prism.oregonstate.edu), while we relied on the 1 × 1 km grid of the German Meteorological Service for the two sites in Bavaria (DWD Climate Data Center, https://opendata.dwd.de/climate_environment/CDC/). We calculated monthly estimates of potential evapotranspiration (PET) using the Hargreaves equation, derived the corresponding climate water balance (CWB = precipitation—PET), and then computed the Standardized Precipitation and Evapotranspiration Index (SPEI) (Vicente‐Serrano *et al*. 2010). The SPEI values were annually integrated over a period of 6 months for the sites in Bavaria (SPEI6, March to August) and 12 months for the sites in Arizona (SPEI12, previous year August to current year July) from 1980 to 2022 (Arizona), respectively to 2023 (Bavaria), to obtain indications of possible dry conditions in certain years. We chose different integration periods to account for the contrasting climate conditions in the two regions. For the USA sites, we referred to a time period that: (i) showed the highest correlations between SPEI and tree growth at our sites (Fig. S2), (ii) that encompassed the region's characteristic bimodal precipitation regime by covering both the winter snowpack and the NAM, which are crucial for tree growth (Bailey *et al*. 2023; Strange *et al*. 2023), and (iii) that spanned the growing season of ponderosa pine and Gambel oak. For the German sites, we chose the period between spring and summer, (i) as this period exhibited the highest correlation between SPEI and tree growth for both species (Fig. S2), and (ii) comprise the main phase of cambial activity of oak (Puchałka *et al*. 2017) and pine (Gruber *et al*. 2010). In accordance with recent observations (Rakovec *et al*. 2022), 2018 and 2022 were the most recent years in which the trees at the Bavarian sites experienced extreme drought (SPEI6 < −2). An exceptionally dry spring and early summer characterized the drought in Central Europe in 2018 (Schuldt *et al*. 2020), while the extreme drought in 2022 lasted for the entire growing season, from April to October (Faranda *et al*. 2023). Arizona, on the other hand, had an exceptionally low winter snowpack in 2018 (Bailey *et al*. 2023), while the late summer of 2020 and the subsequent winter of 2020–2021 were extremely dry (Mankin *et al*. 2021). Thus, both regions experienced recurrent severe drought periods in recent years (2018/2022 for Bavarian sites, 2018/2020–2021 for Arizona sites). We relied on these most recent drought events for further analyses, assuming that the stand conditions (competition, species admixture) have not changed considerably since then.

### Relative growth changes related to recurrent droughts

We quantified the growth changes to the recurrent droughts (i.e. drought responses) following a similar approach to Lloret *et al*. (2011) and Schmied *et al*. (2023). This was defined as follows:

$$\text{Relative}\;{\text{growth change}}_{\mathrm{ij}}\;\left(\%\right)=\frac{{\mathrm{Dr}}_{\mathrm{ij}}}{{\text{PreDr}}_{\mathrm{ij}}}x\;100$$

where ${\mathrm{Dr}}_{\mathrm{ij}}$ indicates the growth (rwi) within the drought year j of target tree i, while ${\text{PreDr}}_{\mathrm{ij}}$ reflects the average growth in a preceding 2‐year period. A 2‐year period was chosen (i) as we found a strong autocorrelation (>0.5) of annual growth for 1‐ to 2‐year lags (Fig. S3) and (ii) because there were at least 2 years between the considered drought years.

### Competition and species admixture

**Fig. 2:**
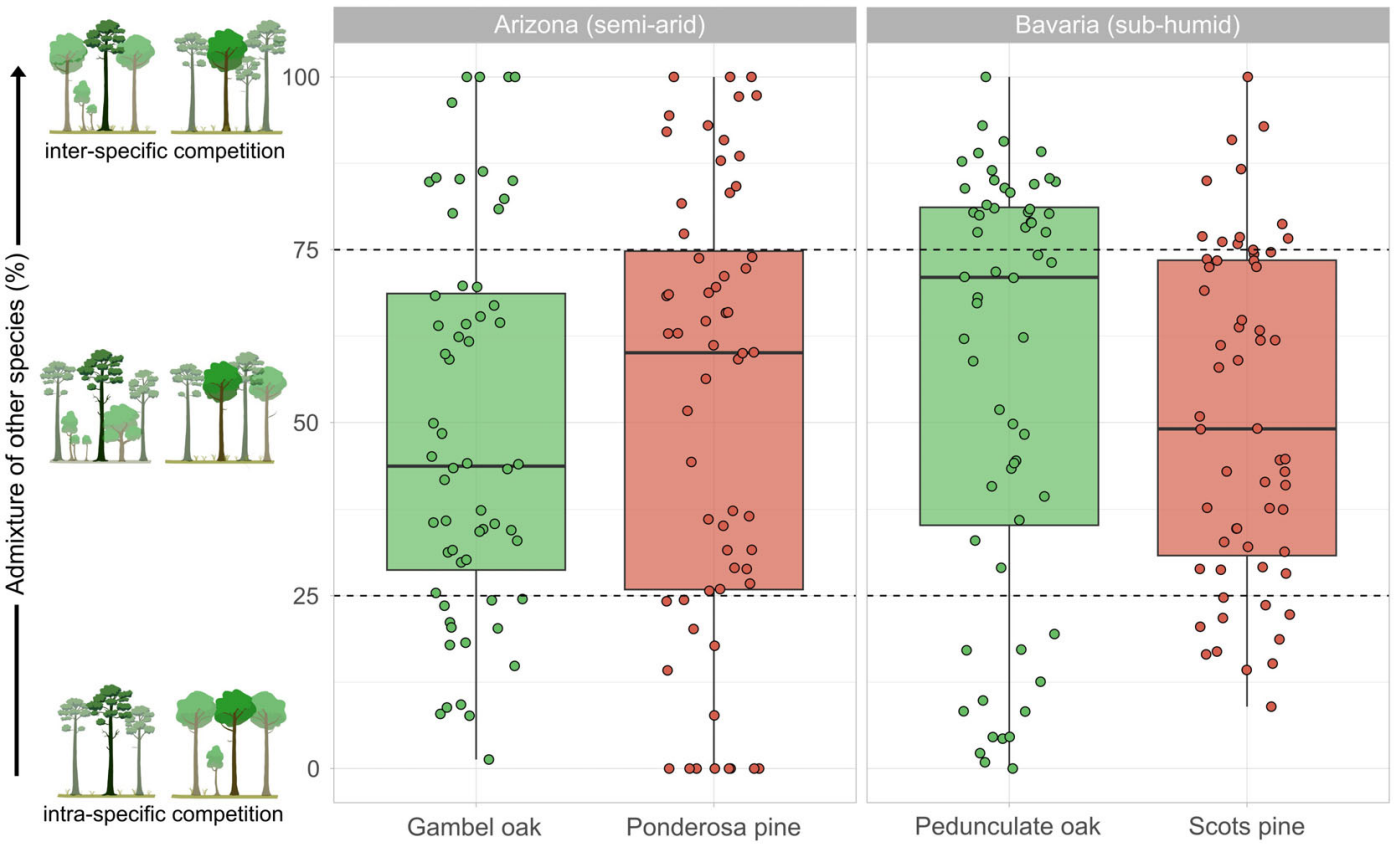
Evaluation of the approach to cover different mixture proportions. Each point represents the competition‐based mixture proportion within the biogroup of a target tree. Dashed lines refer to the thresholds of the initial categories used for tree selection.

The measurements of the neighbouring trees from the 154 m^2^ plots (= biogroups) around each target tree were used to derive overall competition using the distant‐dependent competition index (CI) after Hegyi (1974). The CI is calculated as:

$${\mathrm{CI}}_{i}=\sum_{j=1}^{n}\frac{{\mathrm{dbh}}_{j}}{{\mathrm{dbh}}_{i}}\times \frac{1}{{\text{dist}}_{\mathrm{ij}}}$$

where i is the target tree and j is one of its j=1…n potential competitors, with a dbh_1.3_ ≥ 1 cm situated within the respective biogroup. A higher CI value represents higher competitive pressure on the target tree. As we were particularly interested in determining the actual mixing proportions around each target tree, we additionally calculated the CI as interspecific competition by including only neighbouring trees of species other than the target tree. Subsequently, we assessed the proportion of interspecific competition to overall competition as a measure of the admixture of other species:

$${\text{admixture}}_{i}=\frac{\mathrm{CI}\_{\text{interspecific}}_{i}}{\mathrm{CI}\_{\text{overall}}_{i}}\times 100$$

where i is the target tree. Higher values for admixture (%) indicate a higher proportion of other species among the competitors. An evaluation of the competition‐based mixture proportions for each target tree is given in Fig. 2, confirming the suitability of the tree selection approach and the coverage of a broad mixture gradient.

### Statistical analyses

We built generalized linear mixed effect models (GLMM) within a Bayesian modelling framework to decipher the effects of mixture on drought responses of co‐occurring pine and oaks in the two contrasting forest ecosystems. We have opted for a Bayesian approach as this allows for the consideration of prior information and parameter uncertainty (McElreath 2020). Before modelling, we checked for multi‐collinearity among all predictor variables, relying on correlation coefficients and variance inflation (VIF). The latter was <3 for all considered predictors. All predictor variables were standardized prior to modelling to compile reasonable priors and obtain reliable approximations of the posterior. The standardization of predictors also enabled a direct comparison of regression coefficients (Schielzeth 2010). We used weakly informative priors (mean = 0, SD = 1) to restrict all parameters to a reasonable range and assessed their plausibility by prior predictive checking (Gabry *et al*. 2019; McElreath 2020).

We built GLMMs with no overall intercept and a log‐normal distribution to model the trees' individual response to repeated droughts (= relative growth changes) as a function of (i) admixture (mix), (ii) competition (CI), (iii) tree size (dbh), and (iv) drought timing (first or second drought), incorporating a two‐way interaction between mix and drought. The relative growth changes were modelled separately for each tree species in each region (four models in total). To account for potential differences in environmental conditions and autocorrelation within sites and trees, we included the trees nested in sample sites as varying effects. The model structure was as follows:

$$y\sim \text{lognormal}\;\left(\mu_{i},\sigma \right)$$

$$\mu_{i}=\begin{array}{l} \beta_{\mathrm{CI}}{\mathrm{CI}}_{i}+\beta_{\mathrm{mix}}{\mathrm{mix}}_{i}+\beta_{\mathrm{dbh}}{\mathrm{dbh}}_{i}+\beta_{\text{drought}}{\text{drought}}_{i}\\ +\;\beta_{\mathrm{mix}:\text{drought}}{\mathrm{mix}}_{i}:{\text{drought}}_{i}+x_{\text{Tree}\left[i\right]}\sigma_{\alpha }+x_{\text{Site}\left[i\right]}\sigma_{\gamma } \end{array}$$

where y is a tree's drought response following a log‐normal distribution depending on $\mu_{i}$ and σ. $\mu_{i}$ is then estimated as a function of the fixed effects of β, including varying (random) effects x that account for the nesting of trees within sites.

For Bayesian inference, we relied on Markov Chain Monte Carlo (MCMC) implemented in the probabilistic programming language STAN (Carpenter *et al*. 2017). All models were run with four chains, each with 6000 samples and a warm‐up of 2000 iterations to achieve robust convergence of joint posteriors. Model performance was evaluated by posterior predictive checking to assess discrepancies between observed and simulated data given the fitted model (Gabry *et al*. 2019). In addition, we considered the Bayesian *R* ^2^ as an indicator for goodness‐of‐fit. The convergence of MCMC was checked with the help of trace plots and the Gelman‐Rubin statistic ($\hat{R}$). To assess the effects of potentially influential observations, we applied Pareto‐smoothed importance sampling cross‐validation (PSIS) (McElreath 2020). For the pedunculate oak model, we identified one highly influential tree at the ‘Steinbachwald’ site using PSIS (with *k* = 2.65), which was most likely due to a measurement error. When the tree in question was included in the analysis, the model could not predict the observed data distribution because it was a large outlier. Therefore, we removed this single tree from our analysis.

The statistical environment R, v. 4.3.2, was employed for all analyses (R Core Team 2022). We utilized the packages tidyverse (Wickham *et al*. 2019) for data wrangling; brms (Bürkner 2017) for statistical analyses; bayesplot (Gabry & Mahr 2017) and ggplot2 (Wickham *et al*. 2019) for visualizations; dplR for tree‐ring data processing (Bunn 2008); SPEI (Beguería & Vicente‐Serrano 2017) and prism (Hart & Bell 2015) for working with climate data; and rnaturalearth (South 2017) and sf (Pebesma *et al*. 2022) for handling spatial data.

## RESULTS

### Tree growth and drought responses

**Fig. 3:**
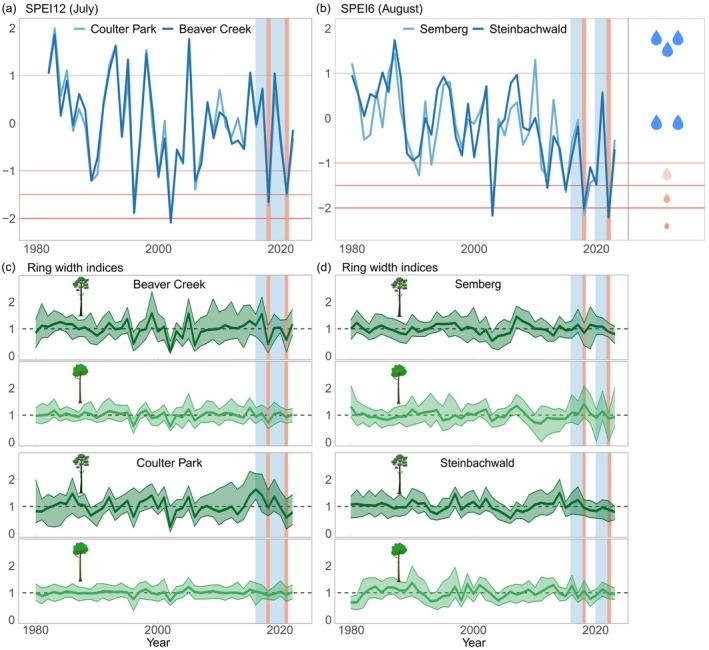
Development of the annual SPEI values for the four sites in Arizona (a) and Bavaria (b), as well as the corresponding detrended growth trajectories (c, d), separated by tree species. Red vertical bars indicate the most recent identified drought years, whereas blue ribbons indicate the reference periods used to derive each tree's individual drought tolerance.

**Fig. 4:**
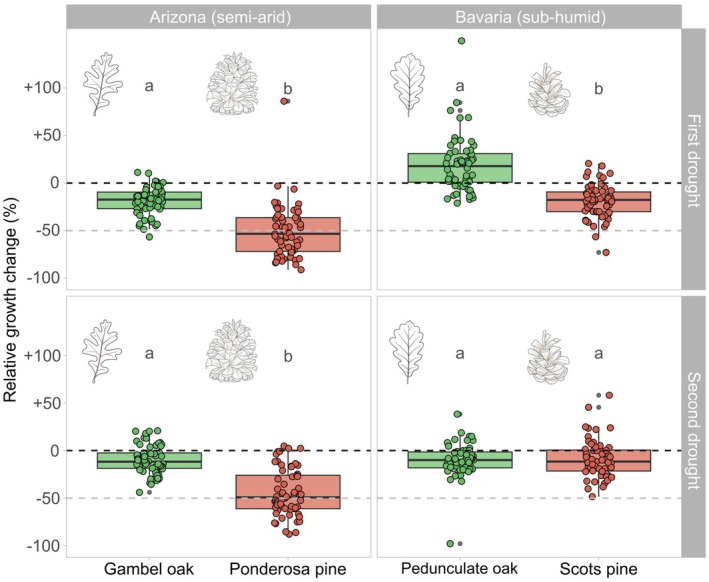
Relative growth change of each species in response to the first (in 2018) and second (in 2020/21 in Arizona and 2022 in Bavaria) considered drought that occurred recently at the study sites. Dashed lines serve as reference lines for no response (black) or a strong (−50%) decline in growth (grey). Different letters refer to pairwise comparisons between tree species per region and drought event, assessed with non‐parametric Wilcoxon rank sum tests with Bonferroni correction. Species that do not share the same letter were significantly different (*P* ≤ 0.001).

For all species, we noticed a high congruence of annual growth patterns (glk ≥0.62) and a strong common signal (rbar 0.32–0.59; eps ≥0.93) among trees from the same site (Table 2). Annual tree growth and climate conditions showed synchronous patterns in both regions, particularly in the past decade (Fig. 3). We observed much higher annual growth rates for ponderosa pine than for Gambel oak in Arizona over the past four decades (Fig. S4, Table 2), with more distinctive growth fluctuations for ponderosa pine (Fig. 3 and Fig. S4). It is noteworthy, however, that the Gambel oaks were generally older than the ponderosa pines (Table 2), reaching an age of up to 300 years (the oldest tree was dated back to 1722). At both sites, the trees experienced major droughts over the past four decades, with significant growth setbacks observed most recently in 2018 and 2021 (Fig. 3), while displaying similar responses to earlier severe droughts, as in 2002 (Fig. S5). In response to the dry conditions in 2018 and 2021, caused by a low winter snowpack and an already dry previous year, both species declined in growth, which was repeatedly more pronounced for ponderosa pine (Fig. 4).

**Table 2:** Overview of growth and chronology characteristics.

site name	tree species	number of trees	DBH (cm)	period (*n* ≥ 5)	mean basal area increment (cm^2^ year^−1^)^a^	rbar^b^	glk^b^	eps^b^
Coulter Park	Gambel oak	30	28.9 ± 6.26	1845–2022	3.46 ± 1.61	0.31	0.62	0.93
	Ponderosa pine	30	35.8 ± 6.68	1921–2022	11.6 ± 7.22	0.54	0.70	0.97
Beaver Creek	Gambel oak	30	29.8 ± 8.44	1844–2022	3.83 ± 1.88	0.38	0.66	0.95
	Ponderosa pine	30	37.9 ± 7.60	1916–2022	10.7 ± 6.89	0.48	0.69	0.97
Semberg	Pedunculate oak	30	35.4 ± 5.46	1914–2023	12.8 ± 5.76	0.59	0.73	0.98
	Scots pine	30	34.5 ± 3.73	1927–2023	11.1 ± 4.75	0.52	0.69	0.97
Steinbachwald	Pedunculate oak	30	30.6 ± 6.31	1980–2023	18.3 ± 10.7	0.42	0.67	0.95
	Scots pine	30	31.2 ± 4.50	1980–2023	18 ± 8.67	0.47	0.67	0.96

eps, expressed population signal; glk, Gleichläufigkeit; rbar, mean inter‐series correlation.

^a^ Based on average values from 1980 to 2022 (2023). Mean ± SD is presented.

^b^ Refers to the period with *n* ≥ 5.

In Bavaria, we observed similar annual growth rates between Scots pine and pedunculate oak, while the younger trees on the more fertile “Steinbachwald” site grew almost twice as much (Table 2). At both sites, there has been a sustained decline in growth of Scots pines in recent years (particularly at the ‘Semberg’ site), which may be related to increasingly dry conditions (Fig. S4, and Fig. 3b), while the oaks have continued to grow at a high rate. A very dry spring and early summer in 2018 led to an immediate growth decline of Scots pines, while most pedunculate oaks showed a contrasting pattern of enhanced growth during that year. However, this changed with the repeated drought in 2022, which was characterized by a very dry growing season. Both species responded similarly to unfavourable climate conditions (Fig. 4).

### Effects of pine and oak admixture in contrasting forest ecosystems

**Fig. 5:**
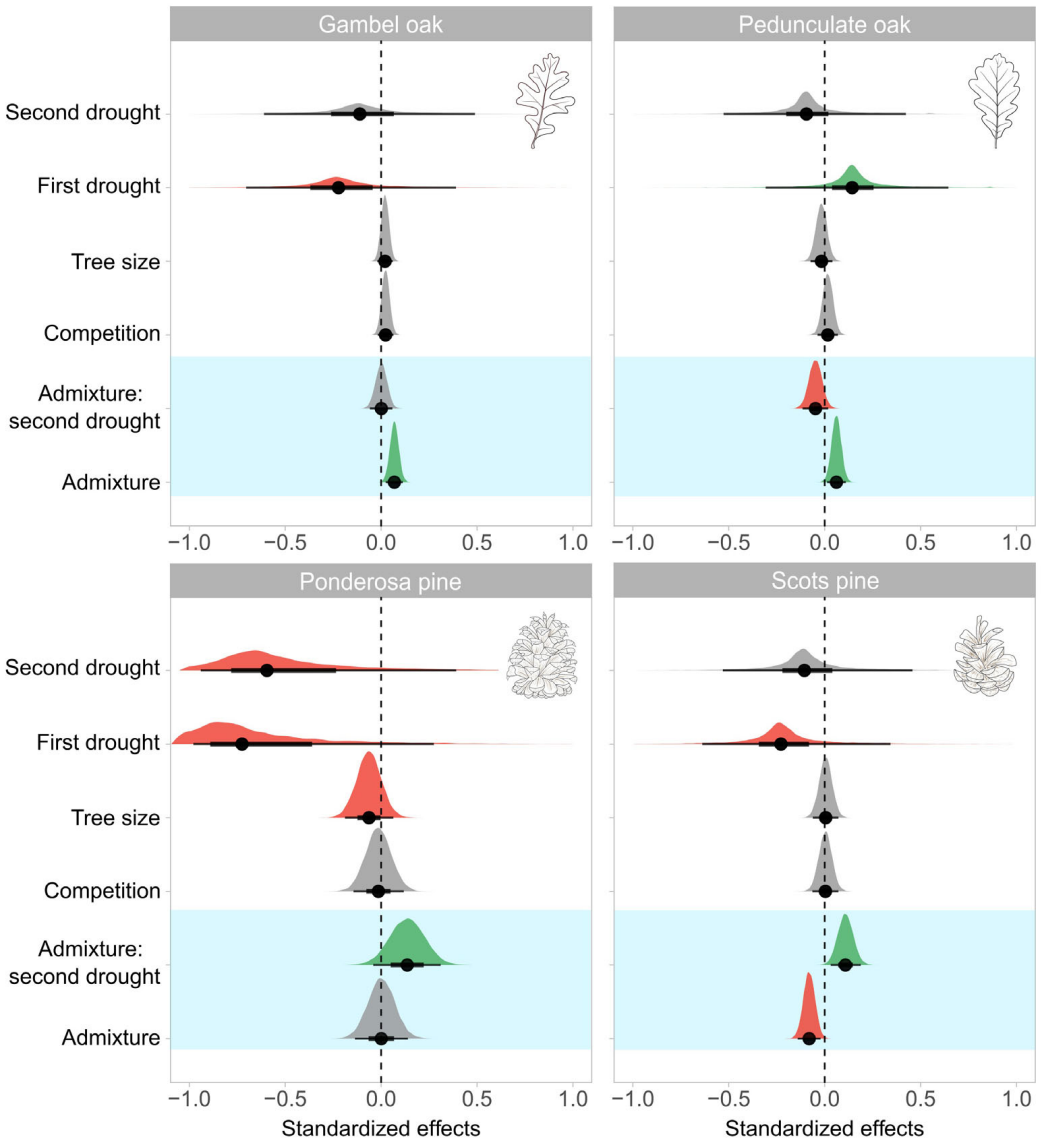
Standardized effects of all considered predictors separately for each modelled tree species (light blue background highlights predictors related to mixture effects). The effects indicate the extent of change in the response variable when the respective predictor is changed by 1 SD. The visualized effect of the interaction ‘admixture: second drought’ is compared with ‘admixture:first drought,’ which serves as reference in the background. The posterior mean (black dots), 66% and 95% credible intervals, and entire posterior distribution are shown. Positive effects are highlighted in green, negative in red and somewhat unclear/neutral effects in grey (= 66% interval tangent to the 0.0 threshold).

**Fig. 6:**
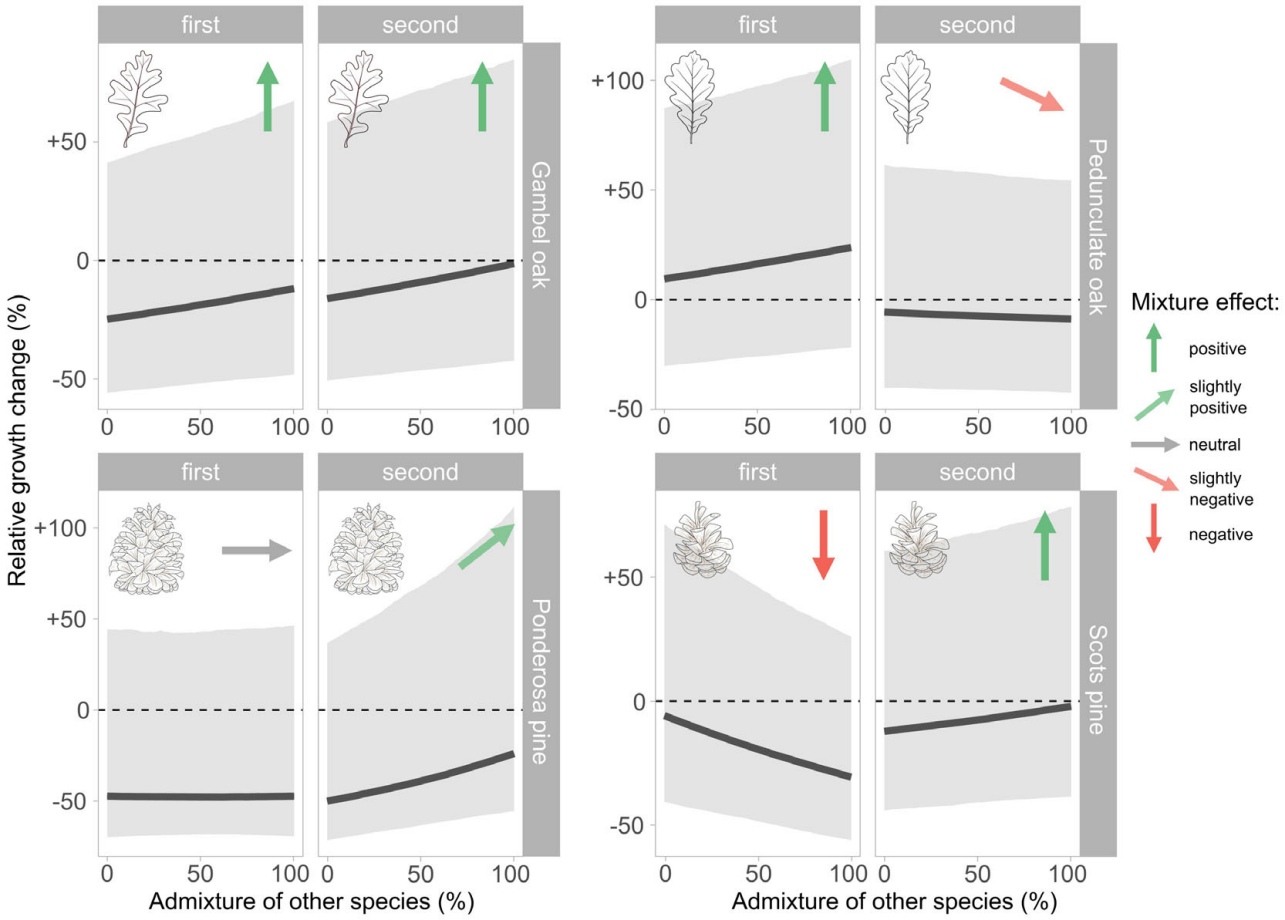
Posterior predicted effects of the interaction between species admixture and drought timing on relative growth change for each modelled tree species. Grey‐shaded areas show the 95% credible intervals, and the black lines show the posterior means. The coloured arrows indicate the general trend based on interpretations of the effects' posterior distributions (see Fig. 5). Note the different scaling on the *y*‐axis.

Species admixture affected the growth responses of pines and oaks in the two contrasting forest ecosystems that experienced recurrent drought stress in recent years (Figs. 5 and 6). Mixture effects differed among tree species and drought timing. In at least one of the two drought periods, each tree species was positively influenced by a higher species admixture (Fig. 6). In semi‐arid Arizona, Gambel oak showed the clearest positive mixture effect, regardless of the drought timing. Here, an increasing admixture proportion reduced the extent of growth reduction caused by drought stress (Figs. 5 and 6). Ponderosa pine trees experienced substantial growth setbacks in both drought years (Fig. 4), with no detectable mixture effect at the first and more severe drought in 2018 (Fig. 6). However, this pattern changed for the second drought event, with a slightly positive admixture effect. In sub‐humid Bavaria, pedunculate oak and Scots pine showed varying mixture effects. Most pedunculate oaks showed enhanced growth during the drought in 2018, which was more pronounced in mixture. In contrast, a slightly negative trend of species admixture was observed during the subsequent, even more extreme drought in 2022 (Fig. 6). The opposite pattern occurred for Scots pine, where growth reduction in response to the first drought increased when growing in mixture, while the pattern was reversed with the second drought. Tree size and competition had little influence on the growth response to drought. Only for ponderosa pine did we observe that larger trees tended to respond more negatively to drought. Despite clear evidence of influence, effect sizes of all predictors (including species admixture) were relatively low, indicating that the mixture influenced the growth responses of the trees but did not substantially change the outcome. Drought timing generated the most prominent effect sizes but with high uncertainty (Fig. 5). For all four species, our models were confident that the variation among sites varied more than among the trees nested within them (Fig. S6). The lowest estimated variation among sites and trees was found for both oak species, while it was highest for ponderosa pine.

The four models exhibited satisfactory performance. The predicted and observed data distributions displayed striking similarity (refer to Fig. S7), confirming that the chosen predictors within the specified model framework successfully predicted various patterns of growth changes to drought. For all models, MCMC convergence was deemed appropriate, as indicated by trace plots and a constant $\hat{R}$ of 1.00. The models accounted for between 26.6% and 35.3% of the total variance.

## DISCUSSION

We utilized mixtures of pine and oak species in two contrasting forest ecosystems to investigate the effects of species admixture under recurrent drought stress. Our overall findings emphasize the positive mixture effects under semi‐arid conditions, most likely due to niche complementarity and facilitative effects, whereas inconclusive patterns were observed under sub‐humid conditions, supporting implications of the SGH hypothesis.

### Niche complementarity explains positive mixture effects in pine–oak forests

Our analysis revealed that admixing ponderosa pine and Gambel oak attenuated drought‐induced growth declines for both species. In contrast, mixtures of Scots pine and pedunculate oak exhibited divergent results, with both positive and negative effects observed. In general, this aligns with previous studies that found positive mixing effects of trees (Fichtner *et al*. 2020; Pardos *et al*. 2021), particularly for species of these genera (Steckel *et al*. 2020). Grossiord (2020) attributed favourable tree species interactions under drought stress to three possible causes: selection effects, resource partitioning and facilitation. In this regard, the dissimilar but complementary traits of pines and oaks can lead to less competition and improved water availability, as their resource use differs in space and time (Poulos 2009; Steckel *et al*. 2020).

Ponderosa pine and Gambel oak are a prime example of this complementarity, which is demonstrated by their distinctive spatial differentiation (see Figs. 1c,d, S1): the evergreen and light‐demanding ponderosa pines occupy the upper canopy layers, while the deciduous and more shade‐tolerant Gambel oaks form a second layer below (Abella 2008). Pine and oak species generally exhibit different crown morphologies, characteristics amplified when growing in mixtures (Pretzsch *et al*. 2020; Hilmers *et al*. 2024). The consequences are increased crown projection area, canopy space‐filling, stand density and growth efficiency (Pretzsch 2014; Pretzsch & Biber 2016; Hilmers *et al*. 2024). These outcomes have positive implications for productivity and can lead to overyielding, particularly in mixtures of evergreen and deciduous species (Lu *et al*. 2016). To return to the example of mixtures of Gambel oak and ponderosa pine, the combination of their different but complementary traits may lead to greater shading and reduced soil evaporation during summer (Breshears *et al*. 1998) and reduced snow ablation rates in spring (Molotch *et al*. 2009; Harpold *et al*. 2015), potentially improving the hydrological conditions throughout the growing season in harsh environments, and thus alleviate drought stress. Trees in the southwestern USA may benefit from a prolonged availability of snowmelt water, because enhanced hydrologic connectivity within the soil also improves water uptake during the summer monsoon (Bailey *et al*. 2023). For ponderosa pines, slowing snowmelt may be crucial as this is their primary water source, especially during the hyper‐arid period (Bailey *et al*. 2023), where the buffer capacity of the NAM is weakening (Strange *et al*. 2023). However, the anticipated overall decline in winter snowpack will likely exacerbate the region's hydrological stress (Gleason *et al*. 2021) and lead to pronounced growth setbacks (Fig. 3).

The niche complementarity of pines and oaks further extends to belowground, where they are often considered to have an inverted pattern, with oaks rooting in deeper soil layers than pines (Steckel *et al*. 2020). Ehleringer & Phillips (1996) highlight the distinct rooting system of Gambel oak that enables it to reach deeper soil layers replenished by winter precipitation, while Bose *et al*. (2021) emphasize the deep‐penetrating roots of pedunculate oak as a favourable trait for drought tolerance. When growing in mixture, a complementary water uptake of pines and oaks was observed during extreme drought (Bello *et al*. 2019), when oaks switch to use of water from deeper layers to which pines have only limited access (Del Castillo *et al*. 2016). This facilitative effect is further consolidated by evidence for redistribution of soil water by Gambel oak and ponderosa pine (Poulos 2009) as well as pedunculate oak (Hafner *et al*. 2017), and is crucial under drought (Hafner *et al*. 2021).

In addition, temporal niche differentiation is considered a contributing factor to resource partitioning in temperate forests, particularly for co‐existing evergreen and deciduous species (Ishii *et al*. 2013). There are indications of such temporal complementarity for some pine and oak species. In semi‐arid Arizona, the bimodal precipitation pattern favours pine and oak growth at different times throughout the year. Oaks photosynthesize more during summer months due to their ability to reach water in deeper soil layers, while the more shallow‐rooted and evergreen pines heavily rely on winter precipitation (Poulos 2009; Bailey *et al*. 2023). In sub‐humid Central Europe, the patterns are less clear or have not yet been thoroughly investigated. More broadly, Larysch *et al*. (2022) revealed that Scots pines are particularly affected by climate conditions in spring, but this was also observed for pedunculate oaks (Bose *et al*. 2021). On the other hand, Muñoz‐Gálvez *et al*. (2021) argued that the divergent water‐use strategies of these species indicate complementary resource use over time, with anisohydric oaks recovering more quickly after drought because of less stringent stomatal control under improved water conditions, while the reduced transpiration of isohydric pines under water stress may alleviate competition for water.

### Persistent positive mixture effects under semi‐arid conditions

We found evidence that mixtures of Gambel oak and ponderosa pine remained beneficial through successive droughts, considering the already dry conditions under the ongoing megadrought in the southwestern USA. Conversely, pedunculate oak and Scots pine mixtures showed inconsistent effects (Fig. 6). Pines exhibited similar growth reductions during both drought events, whereas oaks experienced a growth decline only in response to the successive drought in 2022. While a single drought followed by favourable conditions may enable a tree to recover fully, consecutive droughts occurring before recovery can amplify the overall impact and extend the recovery time (Mitchell *et al*. 2016). Their impacts can accumulate, leading to a lack of hydraulic recovery (Arend *et al*. 2022), hampered leaf development (Kannenberg *et al*. 2019), or depletion of non‐structural carbohydrates (Peltier *et al*. 2023), causing prolonged legacy effects on tree growth (Peltier & Ogle 2019) and vitality (Schmied *et al*. 2023). Pervasive legacy effects are particularly present in dry ecosystems and impair growth and recovery for up to 4 years (Anderegg *et al*. 2015). Szejner *et al*. (2020) demonstrated that recent shifts towards more frequent droughts in the southwestern USA have systematically extended the duration of drought legacies for ponderosa pine. At our sites, both ponderosa and Scots pines have seen sustained growth declines in recent years under exceptionally dry conditions. This is particularly important, as the inability to recover from droughts has been linked to an increased mortality risk in gymnosperms (DeSoto *et al*. 2020). In contrast, this trend was absent in their respective oak species (Fig. S4). This result is consistent with the findings of Anderegg *et al*. (2015), according to which particular species from the Pinaceae family experience considerable legacy effects, although it has been shown that repeated droughts also cause negative legacy effects in oak species (Bose *et al*. 2024). In mixed stands, co‐existing species might adapt to prolonged droughts by adjusting their water uptake to different soil layers, preserving their naturally occurring partitioning of water sources (Grossiord *et al*. 2019), and potentially reducing negative legacy effects. However, Haberstroh & Werner (2022) pointed out that increased interspecific competition can even occur in species mixtures with complementary resource strategies during increasing and extreme drought. This could explain the contrasting mixture effects of pedunculate oak and Scots pine (see Fig. 6). Differences in drought timing could also partly explain these differences, being a limitation of our study. While in semi‐arid Arizona, both drought years (2018 and 2021) were similarly characterized by an exceptionally low winter snowpack, whereas the droughts in sub‐humid Bavaria differed in timing. A dry spring and early summer made 2018 a severe and widespread drought year in Central Europe (Schuldt *et al*. 2020), while the drought in 2022 built up from April to October (Faranda *et al*. 2023), potentially limiting the comparability of the events.

### Different mixture effects across contrasting forest ecosystems

Our study highlights that mixture effects in pine–oak forests during droughts are context‐dependent, with pronounced benefits of species admixtures in semi‐arid Arizona but inconclusive effects in sub‐humid Bavaria. Our results support the findings of Grossiord *et al*. (2014), who revealed that in contrast to grasslands, mixture effects in forests are less clear, depending on the drought exposure of the ecosystem. According to this study, the benefits of a higher species diversity are only present in drought‐prone environments, which is emphasized by findings that complementarity effects increase with decreasing water availability (Forrester 2014), attributing this to the pronounced positive mixture effects of Gambel oak and ponderosa pine in our study. Hence, this aligns well with the theoretical implications of the SGH hypothesis, assuming that positive interactions among plants become more critical when environmental stress increases (Bertness & Callaway 1994). Considering the refinements of the SGH hypothesis suggested by Maestre *et al*. (2009), plant interactions depend on the interacting species' stress tolerance and competitive ability. In the case of the pine–oak mixtures in semi‐arid Arizona, both species show a pronounced drought tolerance and are well adapted to the prevailing conditions. They have evolved a variety of plant functional traits that enhance their survival in high light and low moisture environments (Poulos 2009), with Gambel oak being more drought tolerant and slow growing and ponderosa pine being faster growing and more competitive (see Fig. 4 and Fig. S4). In combination with their spatial and temporal complementarity, this potentially explains their facilitative interactions. In contrast, pedunculate oak and Scots pine in Bavaria reach similar growth levels, which may cause competitive interactions to prevail, even though shifts of their allometric traits appear to increase their complementarity to some degree when grown in admixture (Pretzsch *et al*. 2020; Hilmers *et al*. 2024). As shown in other ecosystem types (e.g. grasslands), drought stress may act as an environmental filter that promotes niche differentiation (Martínez‐Blancas & Martorell 2020). Thus, the stronger complementarity of Gambel oak and ponderosa pine found here could result from stronger selection for niche complementarity between these species in response to the harsher conditions in semi‐arid Arizona.

### Implications for climate‐smart forest management

In the face of climate change, diversifying forests is an important policy to improve resilience and maintain multifunctionality (Messier *et al*. 2022). As pine–oak forest ecosystems occur across the northern hemisphere and often inhabit water‐limited sites, this complementary species mixture is a potential option for climate‐smart forest management (Pretzsch *et al*. 2020). Although our findings provide evidence that mixing pines and oaks can influence growth and potentially buffer against drought stress, these effects are context‐dependent and vary between species and regions, leading us to a similar conclusion as Grossiord *et al*. (2014). In semi‐arid Arizona, fostering natural mixtures of Gambel oak and ponderosa pine could be a viable management option, as their synergistic interactions hold significant potential for improving drought resilience. However, forests of these species are subject to wildfires across their joint distribution range, which is why fire management is essential in these forests. Historical fire exclusion practices reduced the frequency of low‐severity fires, disrupting natural fire regimes and altering forest composition. As a result of fire exclusion and extensive logging of ponderosa pine, Gambel oak densities (particularly of small size) and biomass proliferated, increasing fuel loads and heightening the risk of severe wildfires (Abella & Fulé 2008a; Guiterman *et al*. 2015; Kaufmann *et al*. 2016). Thus, typical treatments strive to reduce Gambel oak densities (Kaufmann *et al*. 2016). While managing fuel loads remains essential to mitigate severe wildfires, the potential benefits of maintaining or even promoting species mixtures warrant reconsideration, particularly given the increasing aridity of the region. Prescribed burning, as demonstrated by Abella & Fulé (2008b), reintroduces or maintains natural low‐severity fire dynamics by selectively reducing smaller Gambel oaks while preserving larger individuals. This approach could balance fuel load reduction with the retention of key Gambel oak structures that may play an important role in alleviating drought stress.

We did not find general positive mixture effects in sub‐humid Bavaria, which differs from other studies in Central Europe on the same species (Pretzsch *et al*. 2020; Steckel *et al*. 2020). In contrast, Pretzsch *et al*. (2020) found over a large scale that Scots pine benefited on rich and oak on poor sites, allowing for a mixture that is productive and stable over a wide ecological gradient, essential under climate change. Although the systematic promotion of mixtures is debatable due to its context‐dependency (Grossiord *et al*. 2014; Haberstroh & Werner 2022; Depauw *et al*. 2024) and also evidence of negative mixture effects (Mas *et al*. 2026), the predicted intensification of drought stress could favour mixed forests, as multiple studies have shown that positive mixture effects become stronger in years with water deficit (Schnabel *et al*. 2019; Fichtner *et al*. 2020). Moreover, mixed forests distribute the risk of being impacted by different stressors among species and size classes, as they tend to respond differently to disturbances, thereby enhancing overall forest stability (Jactel *et al*. 2017). Overall, according to climate‐smart forestry, silvicultural steering to mixed stands with functional complementary species should be fostered on sites that are, or will become, dry, independent of the forest ecosystem.

## AUTHOR CONTRIBUTIONS

**GS:** Conceptualization, methodology, formal analysis, data curation, visualization, investigation, writing—original draft. **JK:** methodology, data curation, investigation, writing—original draft. **MDR:** Supervision, conceptualization, methodology, writing—review and editing. **WKM:** Supervision, conceptualization, methodology, writing—review and editing. **MJG:** Supervision, methodology, writing—review and editing. **TH:** Supervision, methodology, writing—review and editing. **DA:** Investigation, writing—review and editing. **EU:** Methodology, writing—review and editing. **HP:** Supervision, project administration, conceptualization, methodology, funding acquisition, writing—review and editing.

## CONFLICT OF INTEREST

The authors declare no conflicts of interest.

## Supporting information


**Data S1.** Supporting Information.

## Data Availability

Tree ring data for this manuscript are available online at the International Tree‐Ring Data Bank (ITRDB) under the following links (separately for each tree species and study site): Coulter Park—Gambel oak: https://doi.org/10.25921/htzf‐6x59. Coulter Park—Ponderosa pine: https://doi.org/10.25921/dj9m‐8r41. Beaver Creek—Gambel oak: https://doi.org/10.25921/sc15‐yf37. Beaver Creek—Ponderosa pine: https://doi.org/10.25921/h8yw‐7t76. Semberg—Pedunculate oak: https://doi.org/10.25921/reqb‐7a09. Semberg—Scots pine: https://doi.org/10.25921/pbdv‐5439. Steinbachwald—Pedunculate oak: https://doi.org/10.25921/3fqb‐fj43. Steinbachwald—Scots pine: https://doi.org/10.25921/12p5‐te42.
