# Supplementary material for: Positive mixture effects in pine–oak forests during drought are context‐dependent
Source: Plant Biol (Stuttg). 2025 May 7;28(3):950–65. doi: 10.1111/plb.70030 (PMC13089611; doi:10.1111/plb.70030)
Supplement: Supplementary file 1 — Data S1. Supporting Information. [file PLB-28-950-s001.docx]

Supplementary material

Positive mixture effects in pine-oak forests during drought are context-dependent

Gerhard Schmied^1,2*†^, Jonas Kappen^3†^, Miren del Río^4^, W. Keith Moser^5^, Michael J. Gundale^3^, Torben Hilmers^1,2^, Dominik Ambs^1,2^, Enno Uhl^6^, Hans Pretzsch^2,7^

^1^Chair of Tree Growth and Wood Physiology, Department of Life Science Systems, TUM School of Life Sciences, Technical University of Munich, Hans-Carl-von-Carlowitz-Platz 2, 85354 Freising, Germany

^2^Chair for Forest Growth and Yield Science, Department of Life Science Systems, TUM School of Life Sciences, Technical University of Munich, Hans-Carl-von-Carlowitz-Platz 2, 85354 Freising, Germany

^3^Department of Forest Ecology and Management, Swedish University of Agricultural Sciences, SE901-83 Umeå, Sweden

^4^Instituto de Ciencias Forestales ICIFOR-INIA, CSIC, Ctra. A Coruña km 7.5, 28040 Madrid, Spain

^5^USDA Forest Service, Rocky Mountain Research Station, Flagstaff, AZ, USA

^6^Bavarian State Institute of Forestry (LWF), Bavarian State Ministry of Food, Agriculture and Forestry (StMELF), Hans-Carl-von-Carlowitz-Platz 1, 85354 Freising, Germany

^7^Sustainable Forest Management Research Institute iuFOR, University of Valladolid, Valladolid, Spain

*) Corresponding author: gerhard.schmied@tum.de

†) Both authors have contributed equally

1. **Figures:**

Figure S1: Overview of species-specific differences.

Figure S2: Correlation analyses between residual tree-ring chronologies and SPEI at different time scales.

Figure S3: Radial growth autocorrelation.

Figure S4: Species-specific trajectories of basal area increments.

Figure S5: Comparison of responses to previous drought events.

Figure S6: Posterior distributions of the standard deviations of varying effects.

Figure S7: Posterior predictive distributions for each modelled species.


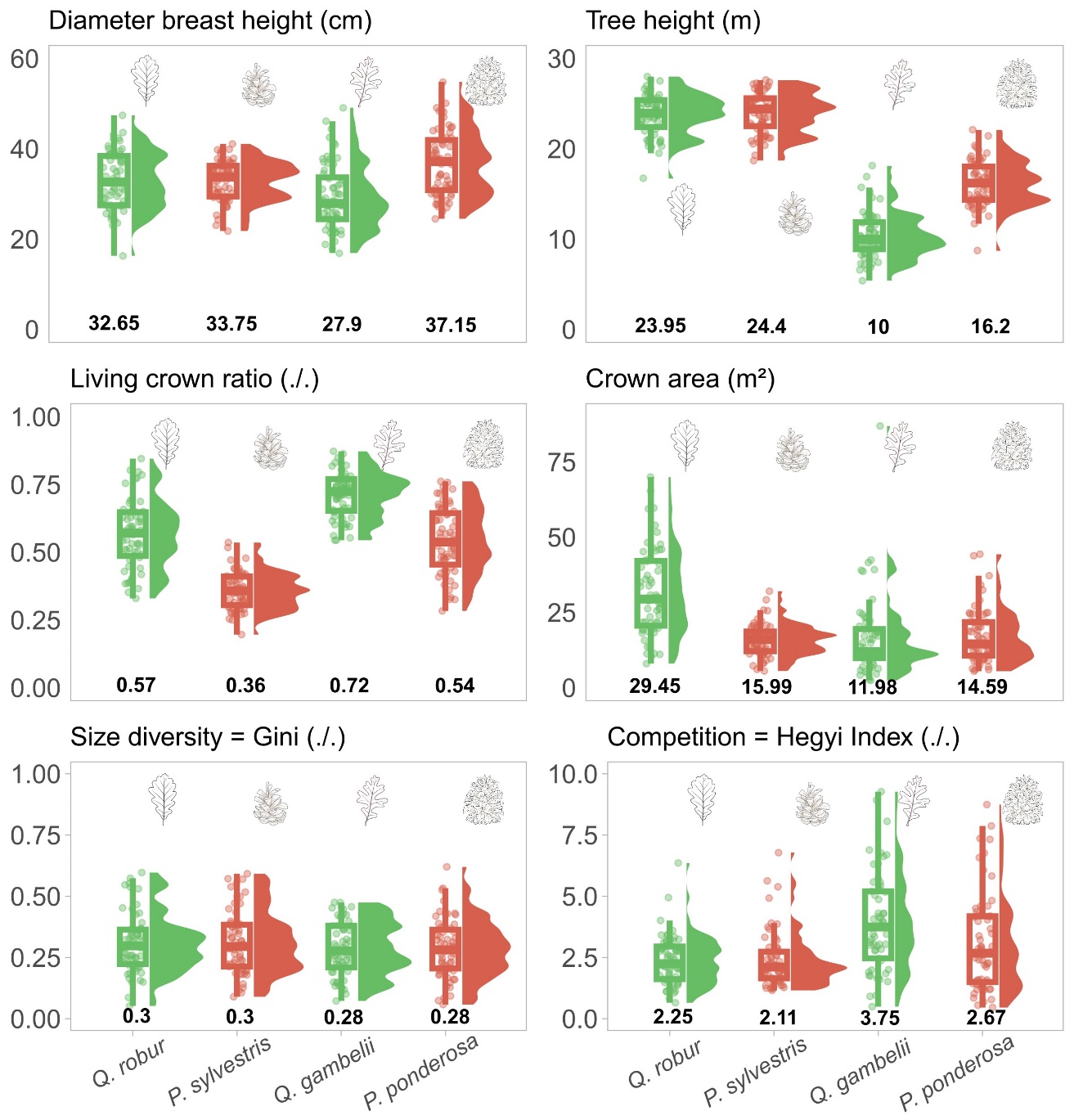


Figure S1: Overview of species-specific differences in dbh, tree height, living crown ratio, crown area, size diversity (gini coefficient of tree sizes), and competition. Pedunculate oak and Scots pine reached similar heights under sub-humid conditions. In contrast, gambel oak consistently had lower heights than ponderosa pine in the semi-arid region – a potential indication of spatial complementarity. This complementarity is also reflected by higher living crown ratios for both species. However, pedunculate oaks in Bavaria formed much larger crowns than gambel oaks, which often occur in clusters with high competition and have slender but long crowns.


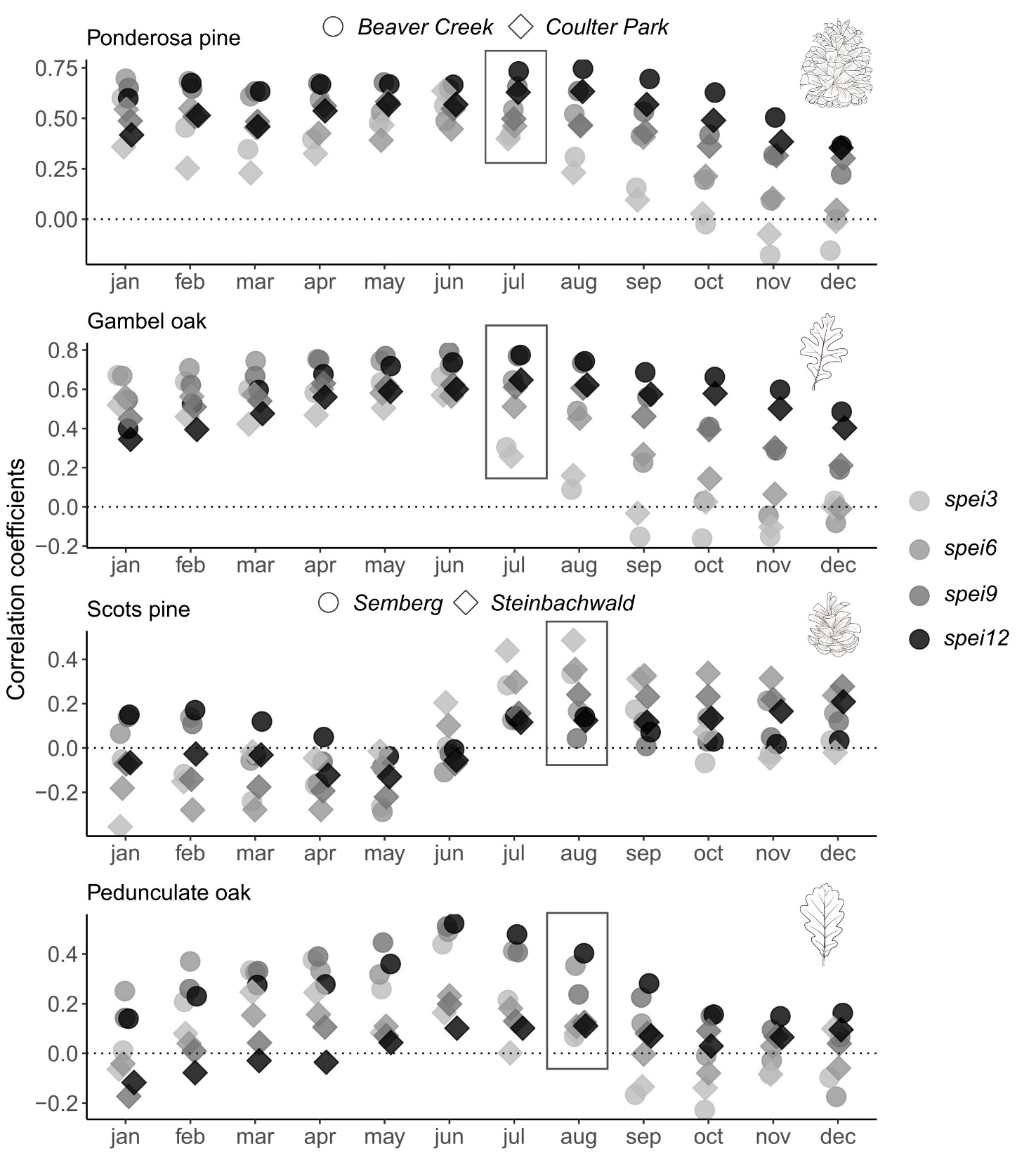


Figure S2: Correlation analyses between residual tree-ring chronologies for each species and SPEI at different time scales (3, 6, 9, or 12 months). The highest Pearson correlations were observed in early summer with long aggregation periods (SPEI12) for ponderosa pine and gambel oak, while the periods with the highest correlations in Bavaria were different for Scots pine and pedunculate oak. A reasonable compromise was to choose SPEI6 in August (see chapter 2.4 in the manuscript). The boxes mark the months that were finally selected (SPEI12 in July for ponderosa pine/gambel oak and SPEI6 in August for Scots pine/pedunculate oak).


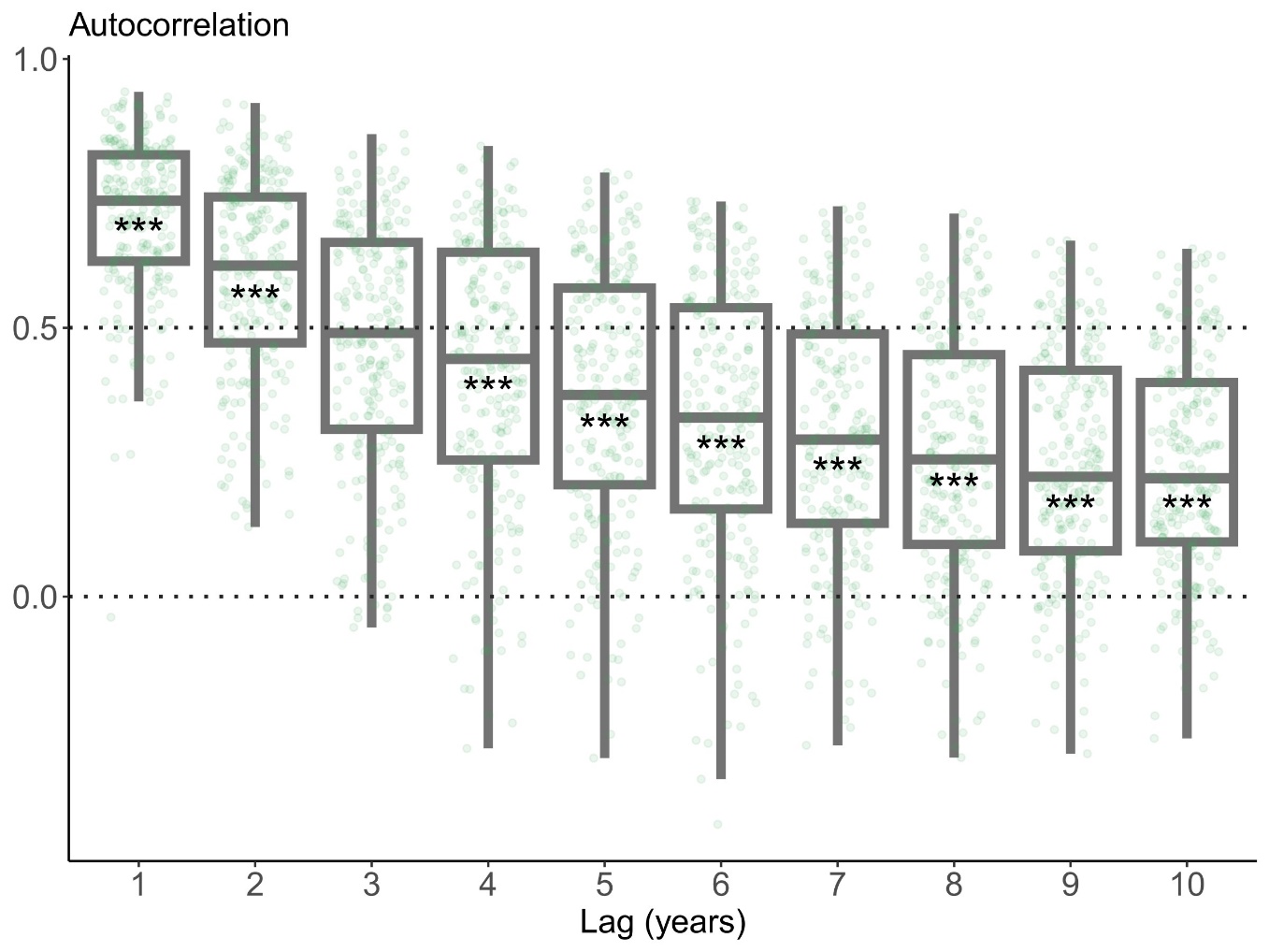


Figure S3: Radial growth autocorrelation for all species combined. Differences between the respective lag and a correlation threshold of 0.5 were tested (ANOVA tests with a post-hoc Tukey Honest Significant Differences). Significance levels: *** = 0.001, ** = 0.01, * = 0.5, ° = 0.1.


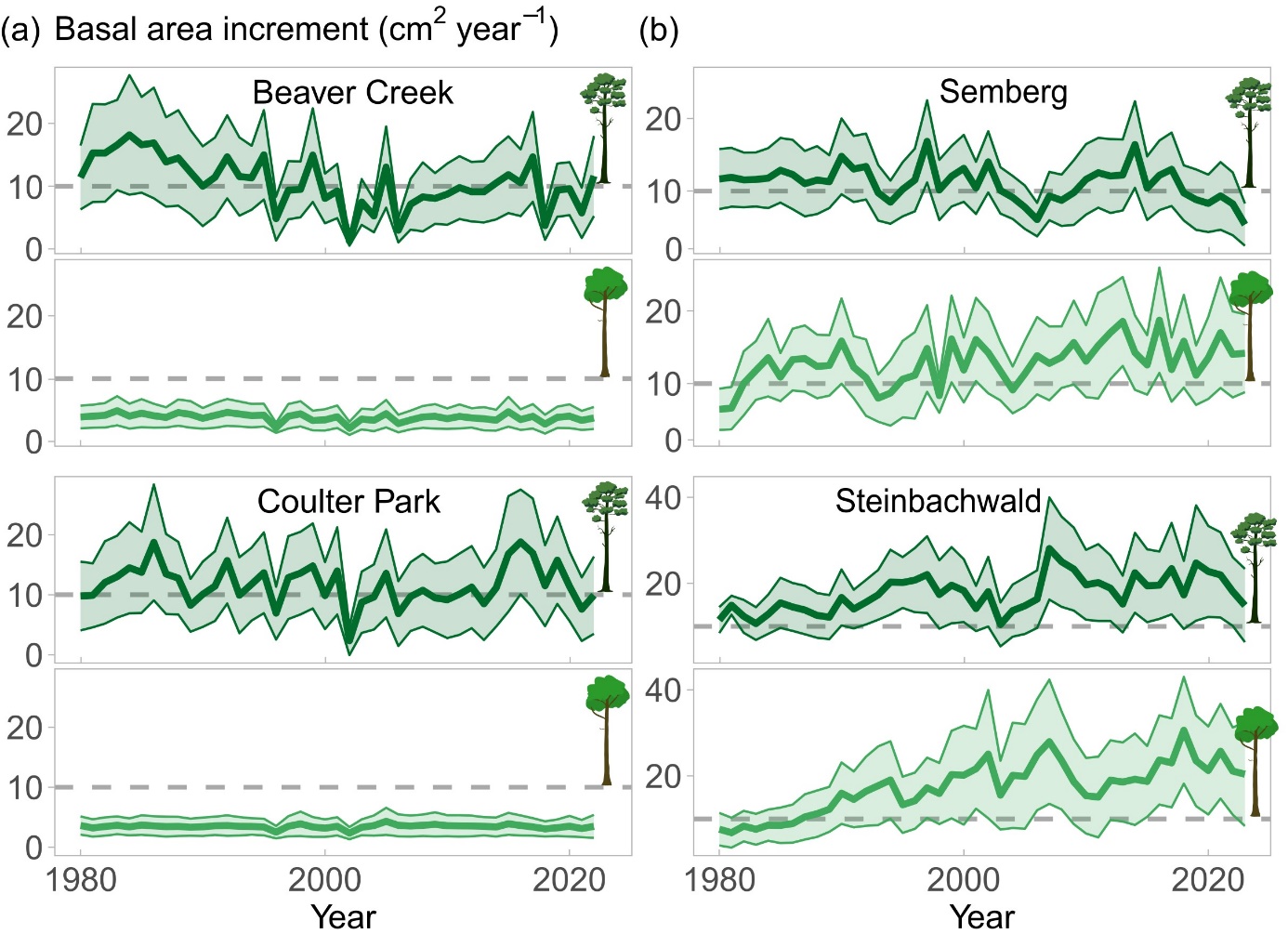


Figure S4: Species-specific trajectories of basal area increments between 1980 and 2023 for Arizona (a) and Bavaria (b). Note the different scaling at the Bavarian site “Steinbachwald.” The grey dashed line functions as a reference line for comparison between the different sites and species.


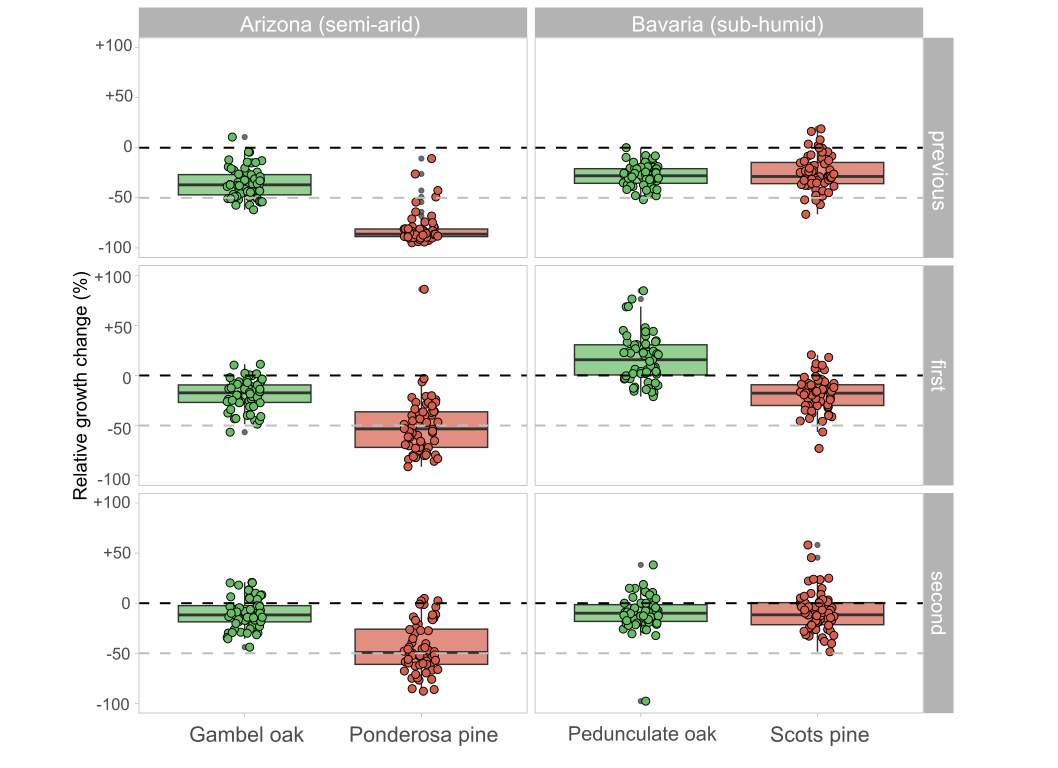


Figure S5: Comparison of responses to past drought events excluded from the analyses due to uncertainties in past mixture proportions. Responses are shown for semi-arid Arizona (reference year: 2002) and sub-humid Bavaria (reference year: 2003), revealing patterns similar to those observed in subsequent drought events.


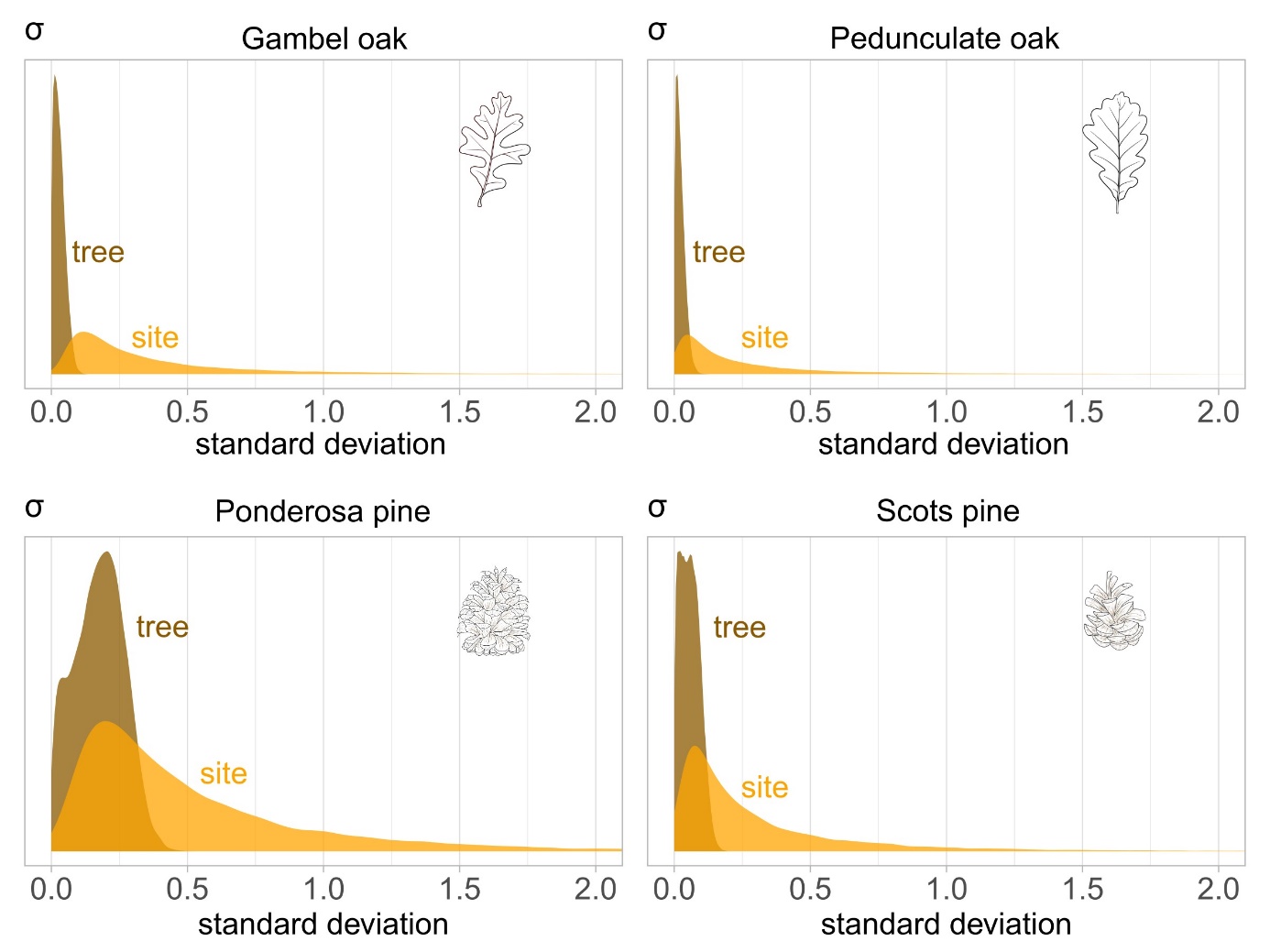


Figure S6: Posterior distributions of the standard deviations of varying tree (darker orange) and site (brighter orange) effects for each individually modelled tree species.


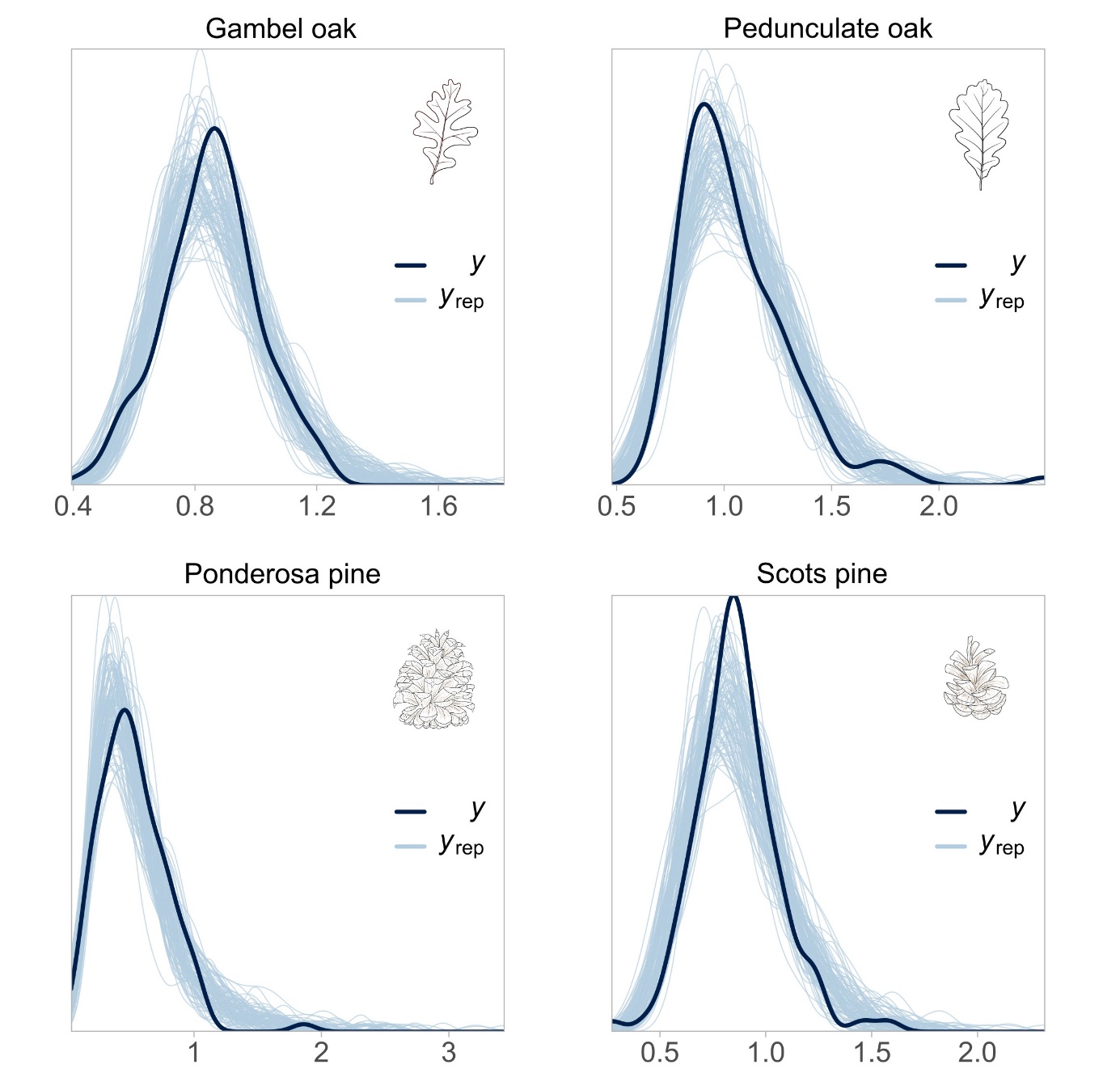


Figure S7: Posterior predictive distributions of the Bayesian multilevel models for each modelled tree species. The observed data distribution ($y$) is highlighted in bold, while the additional lines ($y_{rep}$) indicate 100 data distributions simulated from the respective model. The x-axis depicts the range of the observed data.
